# A Study on the Installation of Facilities for People with Disabilities in Dental Institutions of Chungbuk, South Korea

**DOI:** 10.3390/ijerph181910425

**Published:** 2021-10-03

**Authors:** Sun-ju Kim, Jin-sun Park

**Affiliations:** 1 Department of Dental Hygiene, College of Health & Medical Sciences, Cheongju University, Cheongju 28503, Korea; 2 Dental Center, Hankook General Hospital, Cheongju 28713, Korea; qkrwlstjs46@naver.com

**Keywords:** dental institution, people with disabilities, convenience facilities

## Abstract

To maintain the oral health and ability to smoothly receive dental treatment of people with disabilities, their accessibility to dental institutions must be enforced. Hence, this study aimed to assess the status of installation of convenience facilities for people with disabilities in dental institutions in Chungbuk Province, South Korea. According to the checklist based on installation items for convenience facilities for people with disabilities, 245 dental institutions in Chungbuk Province were visited and investigated to examine whether they had installed internal, intermediary, sanitary, and information-related facilities for people with disabilities. Inputs were analyzed using SPSS 25.0 (IBM, Armonk, NY, USA). Frequency analysis by item was conducted to determine the rate of installation, and Fisher’s exact test was performed to analyze the actual state of installation according to the type of institution. The results revealed a relatively higher rate of appropriate installation and access routes to the main entrance and parking spaces for people with disabilities in terms of intermediary and internal facilities, but at times they were installed at inappropriate locations or lacked proper signboards. The state of installation of convenience facilities by institution type was 0–1.3% per item at the clinic level but 75–87.5% per item in general and dental hospitals (*p* < 0.001). Insufficient government management and supervision at clinic-level dental institutions lead to limited scope for regulating the installation of convenience facilities. Better laws, with continued support and supervision of central and local governments, are needed to resolve this issue.

## 1. Introduction

Convenience facilities for persons with disabilities refer to facilities and equipment that enable convenient—and equal—access to services and information for all people, whether with disabilities or fully fit [1]. These facilities are classified as intermediary facilities, internal facilities, sanitary facilities, and information facilities according to the Enforcement Decree of the Act on Convenience Promotion of people with disabilities, the Elderly and Pregnant Women [2]. They are divided into mandatory and recommended items, with the installation standards stipulated as follows. Secondary medical institutions, such as hospitals, are mandated to install intermediary facilities, internal facilities, sanitary facilities, and information facilities, while primary medical institutions, such as clinics, need to install only intermediary and internal facilities, with toilets only recommended for sanitary facilities. The standards for dental institution convenience facilities mandate that, for intermediary facilities, the pathway from outside to the main entrance of the building should be of an effective width (0.8 m or more), and accessible parking spaces exclusively for persons with disabilities be provided in the ratio specified in the Parking Lot Act. Additionally, if there is a difference in height between the main entrance of the building and the passage, the steps should be lowered, or a wheelchair lift or ramp installed. With respect to internal facilities, the entrances (i.e., doors) and corridors must be of an effective width to allow easy access for those with disabilities. If a medical institution is located in a six-story or higher building with a total floor area of 2000 m^2^ or more, an elevator must be installed. The standards for sanitary facilities include the installation of a washbasin, specifically for people with disabilities, and at least one toilet and one urinal for both males and females. For information facilities, braille blocks should be installed on the sidewalk connecting the building’s main entrance and the road, and braille information boards and alarm systems must be installed near the main entrance.

Although convenience facilities for individuals with disabilities have been progressively improving over the years, they lack quality and are plagued by the persistent problem of limited accessibility [3,4,5]. This makes it inaccessible for people with disabilities when they visit medical institutions, especially preventing access to treatment in a conventional dental clinic setting [6,7,8,9]. People with disabilities are, therefore, reported to have a lower rate of oral examinations and dental treatments and a higher rate of dental caries, periodontal disease, and tooth loss compared with the general population [10,11,12,13,14,15,16,17,18]. Thus, it is necessary to improve accessibility to dental institutions by installing convenience facilities for people with disabilities, to help them maintain healthy oral conditions and receive dental treatment easily. 

According to the 2018 Survey Report on Convenience Facilities for People with Disabilities [19], Chungbuk Province—among all regions in South Korea—showed the lowest rate for both the installation of general facilities and appropriate installations for people with disabilities. To enhance the professionalism and accessibility of dental care services for people with disabilities, the Korean Ministry of Health and Welfare, pursuant to article 15(2) of the Dental Health Act, divided the country into areas and mandated that at least one dental treatment center for persons with disabilities be established as a regional base in each area [20]. Thus, a regional base dental treatment center for people with disabilities is an oral care institution offering services specifically for people with disabilities; it is equipped with facilities and technology that make dental care more accessible and convenient for people with disabilities. As of 2019, Chungbuk is the only province in the country without such an oral health center, or, in fact, any dental institution to provide professional dental care services to persons with disabilities [20].

In this context, this study investigated the current state of installation of convenience facilities for people with disabilities at dental institutions in Chungbuk Province, South Korea, to collect basic data for suggesting appropriate improvements for access and expansion of convenience facilities at such institutions.

## 2. Materials and Methods

### 2.1. Subjects and Methodology

**Figure 1:**
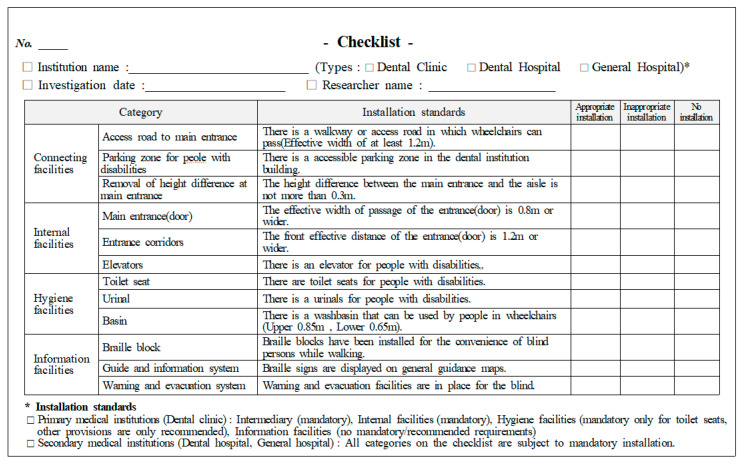
Checklist for installation of convenience facilities.

All dental institutions in Chungbuk registered with the National Health Insurance Service were included as research subjects. A total of 245 dental institutions, registered in Chungbuk as of January 2020, were studied via a complete enumeration survey. To investigate the installation status and standard of convenience facilities for people with disabilities, we visited the dental institutions to record measurements and make observations. Two researchers with previous training in measuring standards prepared a checklist (Figure 1) regarding the amenities for people with disabilities and conducted the investigation. A preliminary examination of 10 medical institutions was conducted to assess inter-investigator reliability. This was followed by Cohen’s kappa analysis, which revealed a kappa value of 0.92 for the internal agreement among investigators, thus indicating a reasonable reliability. The survey period lasted from 1 January to 30 April 2020.

### 2.2. Content of Investigation

The items related to convenience facilities for people with disabilities at dental institutions, as stipulated in Article 4(2) of the Enforcement Decree of the Act on Convenience Promotion of people with disabilities, the Elderly, and Pregnant Women, were analyzed by dividing the area into intermediary facilities, internal facilities, sanitary facilities, and information facilities. From the checklist, under intermediary facilities, the accessibility of the routes to the main entrance, parking zone designated for people with disabilities, and height differences to main entrance (installation of ramps) were investigated; under internal facilities, main entrance (door), corridors, stairs, and elevators installations were examined; under sanitary facilities, toilet seats, urinals, and washbasins for people with disabilities were checked; and for information facilities, the braille block, guide and information system, and warning and evacuation system were examined (Table 1). For each of the facilities, if individual items met the installation standards, it was evaluated as “Appropriate Installation”; if even one item was insufficient, it was evaluated as “Inappropriate Installation”; and if even one item was not installed, the evaluation was “No installation” (Figure 1).

### 2.3. Data Processing and Analysis

The input of the convenience facility checklist was analyzed using SPSS 25.0 (IBM, Armonk, NY, USA). Frequency analysis by item, according to the type of convenience facility, was conducted to determine the rate of installation, and Fisher’s exact test was performed to analyze the actual state of installation according to the type of medical institution. The statistical significance level was set at 0.05.

## 3. Results

### 3.1. General Characteristics of the Investigated Dental Institutions

When grouped according to types of medical institutions examined in this study, dental clinics, which fall under the category of primary medical institutions, formed the majority, with 96.7%, of which 68.6% had been established after 2000 (Table 2).

### 3.2. Installation State of Intermediary Facilities

Table 3 depicts the installation status of intermediary facilities in terms of access route to the main entrance, parking spaces for people with disabilities, and height difference at the main entrance. It was found that most of the access routes to the main entrance were designed just enough to allow wheelchairs to pass. Although the installation rate for disabled parking spaces was high, inappropriate location and inadequate signage were found to be common issues in parking lot design or for entry to the parking lot (Table 3).

### 3.3. Installation State of the Internal Facilities

Table 4 presents the results of the survey on the installation status of internal facilities. Although appropriate installations were high in a majority of the institutions examined, internal facilities require improvement in terms of effective width for corridors and in increasing the installation rate of elevators for people with disabilities (Table 4).

### 3.4. Installation State of the Sanitary Facilities

Table 5 presents the results of the survey on the installation status of sanitary facilities for people with disabilities in the dental institutions examined. Of all the amenities for people with disabilities, the lowest rate of installation was seen among sanitary facilities. In terms of appropriate installation, the rate of installation of toilet seats and urinals for people with disabilities was very low. Bathrooms for people with disabilities were found to be rarely installed, and even if they were installed there were often no separate facilities for men and women, which calls for improvement (Table 5).

### 3.5. Installation State of the Information Facilities

Table 6 presents the installation status of information facilities in the dental institutions. It was found that most medical institutions have alarm and evacuation systems as a part of their information facilities. Nevertheless, because of the low installation rates of braille blocks and guides and information systems for blind persons, there is an urgent need to improve such areas in the future to prevent safety accidents.

### 3.6. Installation State of Convenience Facilities, by Type of Institution

On examining the installation status of convenience facilities by type of dental institution, a significant difference was found between the type of medical institution and the installation status of all facilities (*p* < 0.001). Regarding intermediary facilities, the majority of dental clinics surveyed either did not have them installed or they were installed inappropriately; however, hospital-level dentistry showed a high installation rate of convenience facilities. In medical institutions above the hospital level, internal facilities and information facilities were mostly installed, but they were not installed in any of the dental clinics (*p* < 0.001). This could be because medical institutions at the hospital level or higher are required to create amenities for people with disabilities, while those at clinic level were not required to do so until recently (Table 7).

## 4. Discussion

The basic rights for everyone, including people with disabilities, to use facilities without experiencing any restrictions is known as the rights to access, that is to say, regardless of whether one is a person with disability or able-bodied, all of them have the rights to use facilities without any impediments. The acknowledgement of the accessibility of people with disabilities as ‘rights’ was realized under international norms in the 2006 UN Convention on the Rights of Persons with Disabilities (CRPD) [21]. South Korea signed the UN Convention on the Rights of Persons with Disabilities in 2007, and enforced it with the approval of the National Assembly in 2008. While this was expected to be the impetus to greatly increase the guarantee to human rights and access rights of people with disabilities, it was reported that the domestic efforts to implement the convention were still insufficient [22,23,24]. In addition to the Convention on the Rights of Persons with Disabilities, South Korea stipulates the standards for the installation of convenience facilities for people with disabilities at various institutions and facilities, including medical institution, through the Act on Convenience Promotion of People with Disabilities, the Elderly and Pregnant Women, so as to enhance the access rights of people with disabilities. While ‘accessibility’ signifies the elimination of physical, social, and psychological obstacles for people who seek certain services [24], the term in this study refers to the physical accessibility to facilities and locations that offer dental services. Improving physical accessibility to dental institutions for people with disabilities is the most fundamental and necessary matter, in addition to removing other obstacles such as financial burdens, communication problems, and lack of awareness by medical staff. According to a national survey on the installation status of convenience facilities for people with disabilities at various institutions and facilities, including educational institutions, public institutions and cultural facilities, South Korea’s overall installation rate of convenience facilities for people with disabilities across the country was reported to be low [1,6,19,25,26,27,28,29,30,31]. In order to improve the oral health of people with disabilities, it is imperative to improve their accessibility to specialized dental institutions so that they can receive timely dental treatment immediately when needed, but there is a lack of research on the installation status of convenience facilities for people with disabilities in dental institutions. Therefore, this study was conducted to assess the installation status of convenience facilities for people with disabilities in dental institutions in Chungbuk Province, South Korea.

The findings of this study reveal that the installation rate of convenience facilities for such people at dental institutions in Chungbuk was 2.4%, and the inappropriate installation rate was 97.6%. Therefore, most of the dental institutions investigated in this study were identified as having inappropriate installation of convenience facilities. In a study by Bae [6] on dental hospitals located in Seoul, the overall installation rate of convenience facilities for people with disabilities was 25.8%, which was a quarter of all medical institutions included in the study. The inappropriate installation rate was 66.7% and the non-installation rate was 7.5%. A study by Kim [1] on dental clinics also reported that the installation rate of convenience facilities for people with disabilities is quite low. Investigation of installation conditions of convenience facilities for persons with disabilities by institution type revealed significant differences between hospitals and clinics. It also showed significant differences in terms of the type of medical institution and the installation condition across all convenience facilities (*p* < 0.001). As evident from the results, most of the dental clinics had either no, or inappropriately installed, convenience facilities. However, dental institutions at the hospital level or above were found to have a relatively high rate of installation, which is consistent with the reports of previous studies [6]. Hospitals (including general hospitals, nursing homes, and dental hospitals) are classified as medical facilities under Article 42) of the Enforcement Decree of the Act on Convenience Promotion of people with disabilities, the Elderly and Pregnant Women, which indicates that installation of convenience facilities for people with disabilities is mandatory. However, dental clinics are classified as Type 1 among neighborhood facilities. This means that they have no obligation to install convenience facilities, except when the clinic has an area of 500 m^2^ or more. We assume that this is the reason for the lower installation rate of convenience facilities at clinic-level institutions compared with hospital-level institutions. 

Many domestic studies that examined the installation state of convenience facilities for persons with disabilities in public facilities or organizations reported a state of general insufficiency [25,26,27,28,29,30,31]. Even when installed, the rate of inappropriate installations was quite high. Therefore, it is necessary to strengthen laws and regulations to mandate basic installations of such convenience facilities in most places. Additionally, the mandatory installation standards for these facilities should be further raised, and adequate and appropriate installation should be ensured through continuous supervision.

Owing to poor access to dental service, people with disabilities are likely to postpone or abandon treatment [7,11,12]. They tend to prefer easily accessible dental clinics over general hospitals or dental hospitals. Therefore, legal amendments are necessary to fix the obligation of dental clinics to install convenience facilities for patients with disabilities. In Korea, healthcare and medical needs are considered important. Among the welfare needs of individuals with disabilities, oral healthcare, in particular, is essential—but difficult to access. It was suggested in the study by Rosa et al. [7] that addressing these issues requires training dentists on how to care for people with disabilities, and ensuring that if there are no legal standards governing facilities for people with disabilities, they should be established. Therefore, a revision of laws is required not only for newly established facilities but also for existing ones with inappropriately installed—or missing—convenience facilities; this requires social and economic support from the central and local governments. 

This study is significant because it researched the installation status of convenience facilities for people with disabilities through on-site investigations. These investigations were based on specific criteria to identify the problems that people with disabilities encounter when visiting dental institutions. It also provided the basic data to improve convenience facilities. However, there are limitations in generalizing these research results, as the study was conducted exclusively on dental institutions in one particular region of Korea. Follow-up studies are required to systemically understand the current situation of welfare support and the needs of people with disabilities, to formulate welfare policies for them.

## 5. Conclusions

In conducting this study on 245 dental institutions in Chungbuk Province, South Korea, actual measurements and on-site observations were used to assess the state of installation of convenience facilities for people with disabilities in these institutions. The results of this study and recommendations for improvement can be summarized as follows. 

First, intermediary and internal facilities showed a relatively higher rate of appropriate installation. The rate of installation of access routes to the main entrance and parking spaces for people with disabilities was high, but many were installed at inappropriate locations or lacked proper signboards. Regarding internal facilities, the installation rate for effective width of corridors and elevators for people with disabilities was found to be inadequate.

Second, for sanitary and information facilities, the appropriate installation rate was significantly low. In most of the clinic-level medical institutions—which account for the majority of the medical institutions investigated—toilets for persons with disabilities were either not installed or installed without any distinction between toilets for men and women. Additionally, since the rate of installation of braille blocks and guidance and information facilities for the visually impaired was low, there is an urgent need to improve sanitary and information facilities.

Third, examination of the state of installation of convenience facilities by institution type revealed that the appropriate installation rate at the clinic level was only 0–1.3% per item but was 75–87.5% per item for general and dental hospitals (*p* < 0.001).

Currently, owing to insufficient management and supervision by the Government, the scope to regulate the installation of convenience facilities for people with disabilities at clinic-level dental institutions is limited. Revisions in the laws are required not only for newly established facilities but also for existing facilities with inappropriately installed—or missing—convenience facilities, which requires social and economic support from the central and local governments.

## Figures and Tables

**Table 1:** Type and installation standards of convenience facilities for people with disabilities *.

Type	Installation Standards	Relevant Photos
Road access to main entrance	The access route from outside the facility to the main entrance must be installed considering the effective width, slope, and material and finish of the floor for people with disabilities to pass safely and conveniently (wider than 0.8 m).	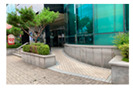
Parking zones for people with disabilities	In the attached parking lot, parking zones for people with disabilities must be designated and located at a place convenient for use in accordance with the installation ratio determined by the Parking Lot Act.	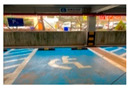
Ramp at the main entrance	If there is a difference in height between the main entrance and the passageway to the building, the height must be lowered or a wheelchair lift or ramp installed (the difference in height must be 0.3 m or less).	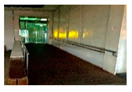
Entrances (doors) that enable access for people with disabilities	The main entrance and at least one of the entrances (doors) of offices intended for public use in the building must be installed considering the effective width, shape, and attachments for people with disabilities to enter (must be 0.8 m or wider).	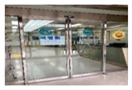
Corridors for people with disabilities	Corridors must be installed considering the effective width, the material of the floor, the finish, and attachments to enable people with disabilities to pass through easily (1.2 m or wider).	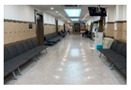
Elevators and escalators for people with disabilities, wheelchair lift	Stairs must be installed in a structure that is convenient for use so that people with disabilities can conveniently move from one floor to another. Alternatively, an elevator, escalator, or a wheelchair lift must be installed for people with disabilities.	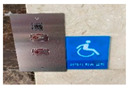
Toilets for people with disabilities	The toilets must be installed after considering the structure, the material of the floor, the finish, and the attachments for people with disabilities to use conveniently.	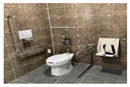
Braille blocks	Braille blocks should be installed on the sidewalk connecting the main entrance of the building to the road or traffic facilities.	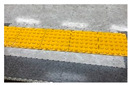
Guide and information system for people with visual or hearing impairment	For people with visual or hearing impairment, at least one braille guide board, tactile guide board, voice guide device, or other guide signal device should be installed near the main entrance of the building in connection with the braille block.	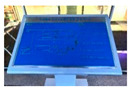
Warning and evacuation systems for people with visual or hearing impairment	To evacuate in an emergency situation, evacuation exit guide lights and passage guide lights must be installed for people with hearing impairments, and warning systems must be installed for people with visual impairments.	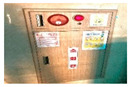

* Article 4 of the Enforcement Decree of the Act on Convenience Promotion of people with disabilities, the Elderly and Pregnant Women.

**Table 2:** General characteristics of the investigated dental institutions.

Type		N	%
Type of dental institution	General Hospitals/Dental Hospitals	8	3.3
	Dental Clinics	237	96.7
Year of establishment	2011 to 2020	93	38.0
	2001 to 2010	75	30.6
	1991 to 2000	59	24.1
	Before 1990	18	7.3

**Table 3:** Installation state of intermediary facilities.

Category	Appropriate Installation	Inappropriate Installation	No Installation
	Frequency (%)	Frequency (%)	Frequency (%)
Access route to main entrance (effective minimum width: 1.2 m)	111 (45.3)	14 (5.7)	120 (49.0)
Installation of parking spaces for people with disabilities	89 (36.3)	0 (0.0)	156 (63.7)
Removal of height difference to main entrance (0.3 m or less height difference)	107 (43.7)	41 (16.7)	97 (39.6)

**Table 4:** Installation state of the internal facilities.

Category	Appropriate Installation	Inappropriate Installation	No Installation
	Frequency (%)	Frequency (%)	Frequency (%)
Entrance (i.e., door) with effective width of 0.8 m or wider	211 (86.1)	14 (5.7)	20 (8.2)
Effective width of corridors 1.2 m or wider	188 (76.7)	0 (0.0)	57 (23.3)
Installation of elevators for people with disabilities	163 (66.5)	5 (2.0)	77 (31.5)

**Table 5:** Installation state of sanitary facilities.

Category	Appropriate Installation	Inappropriate Installation	No Installation
	Frequency (%)	Frequency (%)	Frequency (%)
Toilet seats for people with disabilities	42 (17.1)	0 (0.0)	203 (82.9)
Urinals for people with disabilities	18 (7.3)	0 (0.0)	227 (92.7)
Washbasins for people with disabilities	26 (10.6)	1 (0.4)	218 (89.0)

**Table 6:** Installation condition of the information facilities.

Category	Appropriate Installation	Inappropriate Installation	No Installation
	Frequency (%)	Frequency (%)	Frequency (%)
Braille blocks	8 (3.3)	0 (0.0)	237 (96.7)
Guides and information systems	6 (2.4)	0 (0.0)	239 (97.6)
Alarm and evacuation systems	238 (97.1)	0 (0.0)	7 (2.9)

**Table 7:** Installation conditions of convenience facilities according to the type of medical institutions.

Category	Appropriate Installation	Inappropriate Installation	No Installation	Total	*χ^2^*
	Frequency (%)	Frequency (%)	Frequency (%)		
Dental clinic	0 (0.0)	232 (97.9)	5 (2.1)	237 (100.0)	245.044 ***
Dental hospital	0 (0.0)	2 (100.0)	0 (0.0)	2 (100.0)	
General hospital	6 (100.0)	0 (0.0)	0 (0.0)	6 (100.0)	
Total	6 (2.4)	234 (95.5)	5 (2.0)	245 (100.0)	

Fisher’s exact test, *** *p* < 0.001.

## Data Availability

The data presented in this study are available on request from the corresponding author.
